# Evaluation criterion for response to selection with constraint

**DOI:** 10.1111/asj.13174

**Published:** 2019-02-05

**Authors:** Masahiro Satoh

**Affiliations:** ^1^ Graduate School of Agricultural Science Tohoku University Sendai Japan

**Keywords:** criterion for response to restricted selection, criterion for selecting candidates, restricted BLUP procedure, restricted breeding value, restricted selection index

## Abstract

The aims of the present study are to represent the concept of restricted breeding values algebraically and to propose a criterion for evaluating the genetic responses achieved by using a restricted selection procedure. An additive genetic mixed model characterized by multiple traits with constraints was assumed. If the random errors approach zero and the fixed effects can be completely estimated correctly in the model, the restricted best linear unbiased predictor of breeding values (**u**_R_) is equal to $\left[[I_{q}-G_{0}C_{0}{\left(C_{0}^{\prime }G_{0}C_{0}\right)}^{-1}C_{0}^{\prime }]⊗I\right]u$, where **G**_0_, **C**_0_, and **u** are the additive genetic variance‐covariance matrix for the *q* traits, the matrix for restriction, and the vector of breeding values, respectively. Therefore, if we want to evaluate the response to restricted selection, such as by a stochastic computer simulation study with known breeding values, we can use **u**_R_ as only one criterion.

## 1 INTRODUCTION

Restricted selection is used to control genetic changes in one or more characters that are to be improved. It is a very efficient method of applying antagonistic selection. The concept of imposing restrictions on the conventional selection index was originally proposed by Kempthorne and Nordskog (1959). The index theory was further extended by Harville (1975), Yamada, Yokouchi, and Nishida (1975), and Brascamp (1984) to include constraints on proportional changes. As an extension of the selection index theory, a restricted best linear unbiased prediction (RBLUP) of breeding values (BVs) was developed by Quaas and Henderson (1976). This is accomplished by deriving the best linear unbiased predictors of the BVs, subject to the restriction that the covariance between the predictor and a set of linear functions of the BVs is null.

When we want to improve some traits with constraint, animals are genetically evaluated using a restricted selection index or RBLUP of BVs. However, the concept of restricted selection is obscure and what is estimated on the basis of a restricted selection index or RBLUP procedure is not clear. For example, Díaz, Toro, and Rekaya (1999), and Ieiri, Nomura, Hirooka, and Satoh (2004) used the mean and variance of the achieved genetic response in each of the two traits as their criteria for comparing restricted selection strategies in stochastic simulation studies. Furthermore, if restricted selection is conducted for more than two traits, the comparison becomes more difficult. The objectives of this study are to specify precisely what is estimated on the basis of restricted selection, and to discuss the criteria for selecting candidates and for evaluating the genetic responses achieved when using a restricted selection procedure.

## 2 METHODS

### 2.1 Theoretical background of restricted BLUP procedure

An additive genetic mixed model for *q* traits can be represented as follows:

(1) y=Xb+Zu+e,

where **y** is a vector of observations; **b** is a vector of unknown fixed effects; **u** and **e** are vectors of unknown additive genetic effects and random errors, respectively; and **X** and **Z** are known incidence matrices relating elements of **b** to **y** and **u** to **y**, respectively. Let us assume that **u** and **e** follow a multivariate normal distribution, with $E\left(u\right)=E\left(e\right)=0,var\left(u\right)=G=G_{0}⊗A$, var(e)=R, and $cov(u,e^{\prime })=0$, where $G_{0}$ is an additive genetic variance‐covariance matrix for the *q* traits, **A** is an additive relationship matrix, and **R** is an error variance‐covariance matrix.

Suppose we want to lead a linear function of **b** and **u**, say $w=k^{\prime }b+a^{\prime }u$, in model (1) using a linear function of **y**, say $p^{\prime }y$, where **k** and **a** are any vectors and **p** is some vector for predicting *w*. The predictor, $p^{\prime }y$, is to be chosen such that $E\left(w\right)=E\left(p^{\prime }y\right)$ and $E{\left(p^{\prime }y-a^{\prime }u\right)}^{2}$ is minimally subject to the restriction that $E\left(C^{\prime }u|p^{\prime }y\right)=0,\mathrm{where}\;C^{\prime }\left(=C_{0}^{\prime }⊗I\right)$ is a matrix for restriction (Mallard, 1972). The predictor is

$$p^{\prime }y=k^{\prime }\tilde{b}+a^{\prime }{\mathrm{GZ}}^{\prime }V^{-1}\left[I-\mathrm{ZGC}{\left(C^{\prime }{\mathrm{GZ}}^{\prime }V^{-1}\mathrm{ZGC}\right)}^{-}C^{\prime }{\mathrm{GZ}}^{\prime }V^{-1}\right]\left(y-X\tilde{b}-\mathrm{ZGC}\hat{t}\right),$$

where $\tilde{b}$ and $\hat{t}$ are any solutions to generalized least‐squares equations under a model *E*(**y**) = **Xb** + **ZGCt** and *var*(**y**) = **V** (Quaas & Henderson, 1976). Then the RBLUP of BVs, ${\hat{u}}_{R}$ is

(2) $${\hat{u}}_{R}={\mathrm{GZ}}^{\prime }V^{-1}\left[I-\mathrm{ZGC}{\left(C^{\prime }{\mathrm{GZ}}^{\prime }V^{-1}\mathrm{ZGC}\right)}^{-}C^{\prime }{\mathrm{GZ}}^{\prime }V^{-1}\right]\left(y-X\tilde{b}\right).$$

It can be shown that the predictor is $k^{\prime }\tilde{b}+a^{\prime }{\hat{u}}_{R}$ where $\tilde{b}$ and ${\hat{u}}_{R}$ are solutions to Equation (3):

(3) $$\left[\begin{array}{ccc} X^{\prime }R^{-1}X & X^{\prime }R^{-1}Z & X^{\prime }R^{-1}\mathrm{ZGC}\\ Z^{\prime }R^{-1}X & Z^{\prime }R^{-1}Z+G^{-1} & Z^{\prime }R^{-1}\mathrm{ZGC}\\ C^{\prime }{\mathrm{GZ}}^{\prime }R^{-1}X & C^{\prime }{\mathrm{GZ}}^{\prime }R^{-1}Z & C^{\prime }{\mathrm{GZ}}^{\prime }R^{-1}\mathrm{ZGC} \end{array}\right]\left[\begin{array}{c} \tilde{b}\\ {\hat{u}}_{R}\\ \hat{t} \end{array}\right]=\left[\begin{array}{c} X^{\prime }R^{-1}y\\ Z^{\prime }R^{-1}y\\ C^{\prime }{\mathrm{GZ}}^{\prime }R^{-1}y \end{array}\right].$$

### 2.2 BLUP of BVs with or without missing records

If **e** approaches **0 (e → 0**) in model (1), **y** approaches **Xb + Zu**, then **V** approaches **ZGZ′**. Accordingly we find the vector of predictors ($\hat{u}$) is as follows:

$$\hat{u}={\mathrm{GZ}}^{\prime }V^{-1}\left(y-X\hat{b}\right)={\mathrm{GZ}}^{\prime }{\left({\mathrm{ZGZ}}^{\prime }\right)}^{-1}X\left(b-\hat{b}\right)+{\mathrm{GZ}}^{\prime }{\left({\mathrm{ZGZ}}^{\prime }\right)}^{-1}\mathrm{Zu},$$

where $\hat{b}$ is any solution to the generalized least‐squares equations under a model $E\left(y\right)=\mathrm{Xb};var\left(y\right)=V$. Therefore $\hat{u}$ depends on the estimation accuracy of **b**. It is assumed that only the records of individuals that have records on all the traits or that have no record on any trait are used. Now if **b** can be completely estimated correctly, $\hat{u}$ is

(4) $$\hat{u}={\mathrm{GZ}}^{\prime }{\left({\mathrm{ZGZ}}^{\prime }\right)}^{-1}\mathrm{Zu}=\left[I_{q}⊗{\mathrm{AZ}}_{0}^{\prime }{\left(Z_{0}{\mathrm{AZ}}_{0}^{\prime }\right)}^{-1}Z_{0}\right]u,$$

where **Z**_0_ is the incidence matrix relating the vector of additive genetic effects for each trait to the observations. If records are not missing, **Z**_**0**_ **= I**. Hence $\hat{u}=u$.

Suppose records are ordered by animals within traits, and **u**_0_ and **u**_1_, respectively, are vectors of BVs for animals without and with missing records. Let

$$A=\left[\begin{array}{cc} A_{00} & A_{01}\\ A_{01}^{\prime } & A_{11} \end{array}\right],$$

where the subscripts 0 and 1 of **A** correspond respectively to animals without and with missing records. Accordingly, in an animal model with missing records,

(5) $${\mathrm{AZ}}_{0}^{\prime }{\left(Z_{0}{\mathrm{AZ}}_{0}^{\prime }\right)}^{-1}Z_{0}=A\left[\begin{array}{cc} A_{00}^{-1} & 0\\ 0 & 0 \end{array}\right]=\left[\begin{array}{cc} I & 0\\ A_{01}^{\prime }A_{00}^{-1} & 0 \end{array}\right].$$

Therefore,

(6) $$\hat{u}=\left[I_{q}⊗\left[\begin{array}{cc} I & 0\\ A_{01}^{\prime }A_{00}^{-1} & 0 \end{array}\right]\right]u=\left[\begin{array}{c} u_{0}\\ E(u_{1}|u_{0}) \end{array}\right].$$

where **u**_1_ and **u**_0_ are vectors of BVs for animals with and without missing records. Here, $E\left(u_{i}|u_{j}\right)=\mu_{i}+V_{\mathrm{ij}}V_{\mathrm{jj}}^{-1}\left(u_{j}-\mu_{j}\right)$ is used, where **μ**_*i*_ is the vector of mean of **u**_*i*_ and **V**_*ij*_ is the covariance matrix between **u**_*i*_ and **u**_*j*_.

## 3 RESULTS

### 3.1 RBLUP of BVs imposing the restrictions on all animals

The RBLUP of BVs under mixed model (1) is represented by Equation (2). If **e → 0** in model (1) and **b** can be completely estimated correctly, then the RBLUP of BVs (restricted breeding values: RBVs), **u**_R_, is

$$u_{R}={\mathrm{GZ}}^{\prime }{\left({\mathrm{ZGZ}}^{\prime }\right)}^{-1}\left[I-\mathrm{ZGC}{\left(C^{\prime }{\mathrm{GZ}}^{\prime }{\left({\mathrm{ZGZ}}^{\prime }\right)}^{-1}\mathrm{ZGC}\right)}^{-}C^{\prime }{\mathrm{GZ}}^{\prime }{\left({\mathrm{ZGZ}}^{\prime }\right)}^{-1}\right]\mathrm{Zu}.$$

That is,

(7) $$\begin{array}{cc} u_{R}= & \left[I_{q}⊗{\mathrm{AZ}}_{0}^{\prime }{\left(Z_{0}{\mathrm{AZ}}_{0}^{\prime }\right)}^{-1}Z_{0}-G_{0}C_{0}{\left(C_{0}^{\prime }G_{0}C_{0}\right)}^{-1}C_{0}^{\prime }⊗\right.\\ & \left[{\mathrm{AZ}}_{0}^{\prime }{\left(Z_{0}{\mathrm{AZ}}_{0}^{\prime }\right)}^{-1}Z_{0}A{\left[{\mathrm{AZ}}_{0}^{\prime }{\left(Z_{0}{\mathrm{AZ}}_{0}^{\prime }\right)}^{-1}Z_{0}A\right]}^{-}{\mathrm{AZ}}_{0}^{\prime }{\left(Z_{0}{\mathrm{AZ}}_{0}^{\prime }\right)}^{-1}Z_{0}\right]u. \end{array}$$

Since $\begin{array}{cc} {\mathrm{AZ}}_{0}^{\prime }\left(Z_{0}{\mathrm{AZ}}_{0}^{\prime }\right) & {}^{-1}Z_{0}A{\left[{\mathrm{AZ}}_{0}^{\prime }{\left(Z_{0}{\mathrm{AZ}}_{0}^{\prime }\right)}^{-1}Z_{0}A\right]}^{-}\\ & {\mathrm{AZ}}_{0}^{\prime }{\left(Z_{0}{\mathrm{AZ}}_{0}^{\prime }\right)}^{-1}Z_{0}{\mathrm{AA}}^{-1}={\mathrm{AZ}}_{0}^{\prime }{\left(Z_{0}{\mathrm{AZ}}_{0}^{\prime }\right)}^{-1}{Z^{\prime }}_{0}, \end{array}$ then

(8) $$u_{R}=\left[\left[I_{q}-G_{0}C_{0}{\left(C_{0}^{\prime }G_{0}C_{0}\right)}^{-1}C_{0}^{\prime }\right]⊗\left[{\mathrm{AZ}}_{0}^{\prime }{\left(Z_{0}{\mathrm{AZ}}_{0}^{\prime }\right)}^{-1}Z_{0}\right]\right]u.$$

Equation (10) represent the generalized RBVs imposing the restrictions on all animals. When records are not missing, ${\mathrm{AZ}}_{0}^{\prime }{\left(Z_{0}{\mathrm{AZ}}_{0}^{\prime }\right)}^{-1}Z_{0}=I$. Hence

(9) $$u_{R}=\left[\left[I_{q}-G_{0}C_{0}{\left(C_{0}^{\prime }G_{0}C_{0}\right)}^{-1}C_{0}^{\prime }\right]⊗I\right]u$$

when there are some animals with missing records. From Equations (5), (6), and (10),

(10) $$u_{R0}=\left[\left[I_{q}-G_{0}C_{0}{\left(C_{0}^{\prime }G_{0}C_{0}\right)}^{-1}C_{0}^{\prime }\right]⊗I\right]u_{0},$$

$$u_{R1}=\left[\left[I_{q}-G_{0}C_{0}{\left(C_{0}^{\prime }G_{0}C_{0}\right)}^{-1}C_{0}^{\prime }\right]⊗I\right]E\left(u_{1}|u_{0}\right),$$

where the subscripts 1 and 0 of **u**_R_ respectively correspond to animals with and without missing records.

### 3.2 RBLUP of BVs imposing the restrictions on some animals

If the constraints are imposed on the BVs of some animals in a population, $C=C_{0}⊗J$, where **J** is the identity matrix with columns pertaining to animals without constraints deleted (Satoh, 2004). From Equation (8),

$$\begin{array}{cc} u_{R} & =\left[I_{q}⊗{\mathrm{AZ}}_{0}^{\prime }{\left(Z_{0}{\mathrm{AZ}}_{0}^{\prime }\right)}^{-1}Z_{0}-G_{0}C_{0}{\left(C_{0}^{\prime }G_{0}C_{0}\right)}^{-1}C_{0}^{\prime }\right.\\ & \left.⊗{\mathrm{AZ}}_{0}^{\prime }{\left(Z_{0}{\mathrm{AZ}}_{0}^{\prime }\right)}^{-1}Z_{0}\mathrm{AJ}{\left[J^{\prime }{\mathrm{AZ}}_{0}^{\prime }{\left(Z_{0}{\mathrm{AZ}}_{0}^{\prime }\right)}^{-1}Z_{0}\mathrm{AJ}\right]}^{-}J^{\prime }{\mathrm{AZ}}_{0}^{\prime }{\left(Z_{0}{\mathrm{AZ}}_{0}^{\prime }\right)}^{-1}Z_{0}\right]u. \end{array}$$

Now to simplify we assumed that animals without constraints have records of all traits and let the vector of their BVs be **u**_2_. Then

$$var\left[\begin{array}{c} u_{2}\\ u_{0}\\ u_{1} \end{array}\right]=\sigma_{a}^{2}A=\sigma_{a}^{2}\left[\begin{array}{ccc} A_{22} & A_{20} & A_{21}\\ A_{02} & A_{00} & A_{01}\\ A_{12} & A_{10} & A_{11} \end{array}\right].$$

Let the inverse of $Z_{0}{\mathrm{AZ}}_{0}^{\prime }$ be

$${\left(Z_{0}{\mathrm{AZ}}_{0}^{\prime }\right)}^{-1}={\left[\begin{array}{cc} A_{22} & A_{20}\\ A_{02} & A_{00} \end{array}\right]}^{-1}=\left[\begin{array}{cc} A^{22} & A^{20}\\ A^{02} & A^{00} \end{array}\right].$$

We have

(11) $$u_{R0}=\left[\left[I_{q}-G_{0}C_{0}{\left(C_{0}^{\prime }G_{0}C_{0}\right)}^{-1}C_{0}^{\prime }\right]⊗I\right]u_{0},$$

and

$$\begin{array}{cc} u_{R1} & =\left(I_{q}-G_{0}C_{0}{\left(C_{0}^{\prime }G_{0}C_{0}\right)}^{-1}C_{0}^{\prime }\right)⊗\left(E\left(u_{1}|u_{2}\right)+A_{10}A^{02}u_{2}\right)\\ & +\left(I_{q}-G_{0}C_{0}{\left(C_{0}^{\prime }G_{0}C_{0}\right)}^{-1}C_{0}^{\prime }\right)⊗\left(A_{12}A^{20}u_{0}+E\left(u_{1}|u_{0}\right)\right). \end{array}$$

The proof is shown in the Appendix.

### 3.3 Criteria for selecting candidates and response to restricted selection

From Equations (9) through [Link], a vector of the BVs is transformed into the corresponding vector of the RBV by the transformation matrix $\left[I_{q}-G_{0}C_{0}{\left(C_{0}^{\prime }G_{0}C_{0}\right)}^{-1}C_{0}^{\prime }\right]$. Note that both order of vectors BVs and RBVs is *q*, but the dimensions of the vector spaces generated by BVs and RBVs are *q* and (*q *−* r*), respectively. When the linear model used is Equation (1), the RBLUP of **u**, ${\hat{u}}_{R}$, is

$$\begin{array}{cc} {\hat{u}}_{R} & ={\mathrm{GZ}}^{\prime }V^{-1}\left[I-\mathrm{ZGC}{\left(C^{\prime }{\mathrm{GZ}}^{\prime }V^{-1}\mathrm{ZGC}\right)}^{-}C^{\prime }{\mathrm{GZ}}^{\prime }V^{-1}\right]\left(y-X\tilde{b}\right)\\ & ={\mathrm{GZ}}^{\prime }S\left[I-X{\left(X^{\prime }\mathrm{SX}\right)}^{-}X^{\prime }S\right]y, \end{array}$$

where $S=V^{-1}\left[I-\mathrm{ZGC}{\left(C^{\prime }{\mathrm{GZ}}^{\prime }V^{-1}\mathrm{ZGC}\right)}^{-}C^{\prime }{\mathrm{GZ}}^{\prime }V^{-1}\right]$. Therefore, note that in model (1) ${\hat{u}}_{R}$ is obviously different from the BLUP of $[I-\mathrm{GC}{\left(C^{\prime }\mathrm{GC}\right)}^{-1}C^{\prime }]u$.

Supposing that ${\hat{u}}_{R}$ is the solution vector in Equation (3), then the total merit of the *j* ^th^ animal (${\hat{H}}_{R_{j}}$) is

(12) $${\hat{H}}_{R_{j}}=v^{\prime }{\hat{u}}_{R_{j}}$$

where **v** is the economic weights and ${\hat{u}}_{R_{j}}$ is the RBLUP of **u** corresponding to the *j* ^th^ animal in Equation (3). Then, ${\hat{H}}_{R_{j}}$ is the criterion for selecting candidates on the basis of an RBLUP procedure. However, we cannot use Equation [Link] to evaluate the genetic responses achieved on the basis of restricted selection other than the RBLUP procedure if the vector of BVs is known such as in stochastic computer simulation studies. In this case, Equation [Link] can be used as only one criterion for evaluating responses to selection. Then, the total merit $\left(H_{R_{j}}\right)$ on the basis of the RBVs of the *j* ^th^ animal $\left(u_{R_{j}}\right)$ is as follows:

$$H_{R_{j}}=v^{\prime }u_{R_{j}}=v^{\prime }\left[I_{q}-G_{0}C_{0}{\left(C_{0}^{\prime }G_{0}C_{0}\right)}^{-1}C_{0}^{\prime }\right]u_{j}$$

where **u**_*j*_ is the vector of the BVs of the *j* ^th^ animal. If constraints include zero change in one or a few traits, the elements of $u_{R_{j}}$ corresponding to the restricted traits are 0; and if constraints are proportional changes (including no change) for all traits, the elements of $u_{R_{j}}$ are proportional to the relative desired changes. Note that ${\hat{H}}_{R_{j}}\neq H_{R_{j}}$ in general.

If we want to evaluate responses to restricted selection of the current population, the following equation is useful.

(13) $$H_{R}=v^{\prime }(\sum_{j}^{n_{m}}u_{R_{\mathrm{mj}}}/n_{m}+\sum_{j}^{n_{f}}u_{R_{\mathrm{fj}}}/n_{f})/2=v^{\prime }\left[I_{q}-G_{0}C_{0}{\left(C_{0}^{\prime }G_{0}C_{0}\right)}^{-1}C_{0}^{\prime }\right]\overset{¯}{u},$$

where $u_{R_{\mathrm{mj}}}$ and $u_{R_{\mathrm{fj}}}$ are vectors of RBVs of the *j* ^th^ male and female in the current population, respectively, *n*_*m*_ and *n*_*f*_ are numbers of males and females, respectively, and $\overset{¯}{u}=({\overset{¯}{u}}_{m}+{\overset{¯}{u}}_{f})/2$, where ${\overset{¯}{u}}_{m}$ and ${\overset{¯}{u}}_{f}$ are mean vectors of BVs of males and females, respectively. Even if we do not have any true BVs, we can use phenotypes or BVs of BLUP instead of true BVs in Equation [Link].

### 3.4 Numerical example of RBVs

The results of a Monte Carlo computer simulation by Ieiri, Nomura, Hirooka, and Satoh (2004) were used as a numerical example. In this study, selection for two traits was assumed, in which animals were selected to maximize the genetic gain (Δ*G*_2_) in trait 2 under a proportional restriction on the genetic gain in trait 1 (Δ*G*_1_) to satisfy the intended ratio (Δ*G*_1_:Δ*G*_2_ = −2:1). The heritabilities of the two traits were both 0.3 and genetic and phenotypic correlations between them were both 0.4 (Basic set). A larger population (Set A), two different phenotypic correlations (Set B), and two different biased estimations of generic correlation (Set C) were used in the simulations. Two schemes of restricted selection were conducted in each set. The first selection scheme was the combination of BLUP evaluation and linear programming technique (BLUP + LP) and the second one was based on RBLUP selection.

The left‐hand side of Table 1 shows genetic gains (BVs) selected by BLUP + LP and RBLUP in each set. In this case, there are two values of evaluation of selection with constraints. Then, by using Equation [Link], we evaluate these genetic responses to selection. Because the constraints are proportional changes for all traits, no economic weights are needed. Assuming that the genetic variances of the two traits are both 1.0, then

$$G_{0}=\left[\begin{array}{cc} 1.0 & 0.4\\ 0.4 & 1.0 \end{array}\right],C_{0}=\left[\begin{array}{c} 1\\ 2 \end{array}\right].$$

**Table 1:** Numerical example of the relationship between genetic gains and restricted breeding values for two traits

Set^a^	Genetic gain (BV)				Restricted breeding value (RBV)			
	BLUP + LP^b^		R‐BLUP^c^		BLUP + LP^b^		R‐BLUP^c^	
	Trait 1	Trait 2	Trait 1	Trait 2	Trait 1	Trait 2	Trait 1	Trait 2
Basic	−0.536	0.256	−0.551	0.261	−0.530	0.265	−0.543	0.272
A	−0.556	0.265	−0.561	0.277	−0.549	0.275	−0.559	0.280
B‐1	−0.480	0.225	−0.488	0.236	−0.472	0.236	−0.484	0.242
B‐2	−0.709	0.356	−0.704	0.358	−0.710	0.355	−0.707	0.354
C‐1	−0.557	0.231	−0.606	0.183	−0.531	0.266	−0.541	0.270
C‐2	−0.519	0.281	−0.500	0.324	−0.531	0.265	−0.540	0.270

BLUP, best linear unbiased prediction.

^a^Six different sets of parameters were simulated. See Ieiri et al. (2004) in detail. ^b^Combined selection of ordinary BLUP and linear programming technique. ^c^Restricted BLUP selection.

Hence,

(14) $$H_{R}=v^{\prime }\left[I_{q}-G_{0}C_{0}{\left(C_{0}^{\prime }G_{0}C_{0}\right)}^{-1}C_{0}^{\prime }\right]\overset{¯}{u}=\left[\begin{array}{cc} 0.7273 & -0.5455\\ -0.3636 & 0.2727 \end{array}\right]\overset{¯}{u}.$$

Using Equation [Link], we can obtain RBVs on the right‐hand side of Table 1. Note that the RBVs of trait 1 and trait 2 are proportional to the predetermined proportional changes (−2:1). Consequently, the RBV of a trait is dependent on that of another trait.

## 4 DISCUSSION

Restricted selection is an efficient method of antagonistic selection. Kempthorne and Nordskog (1959) gave the basic derivation of restricted selection indices. As an extension of the selection index theory, Quaas and Henderson (1976) derived the RBLUP procedure by imposing restrictions on multiple‐trait BLUP. Later, Satoh (2018) provided an alternative derivation method of mixed model equations from RBLUP. The original method for calculating RBLUP, imposing the same restrictions on all animals has been studied (Itoh & Iwaisaki, 1990; Quaas & Henderson, 1976; Satoh, 1998). Satoh (2004) indicated a new procedure for computing RBLUP of BVs when constraints are imposed on the BVs of only some animals in a population.

On the other hand, another restricted selection procedure using linear programming (LP) techniques has been proposed as an alternative to the restricted selection index for sire selection (Famula, 1992). The efficiency of selection with LP techniques has been compared with RBLUP selection in simulation studies (Díaz et al., 1999; Ieiri, Satoh, & Murakami, 1996; Ieiri et al., 2004; Toro & Silió, 1992). In those studies, the mean and variance of the achieved genetic response in each of the two traits were employed as criteria for comparing restricted selection strategies. However, if restricted selection for more than two traits is conducted in a simulation study, the comparison is more difficult.

In any case, BVs in each animal with constraints are linearly transformed to RBVs by the transformation matrix $I_{q}-G_{0}C_{0}{\left(C_{0}^{\prime }G_{0}C_{0}\right)}^{-1}C_{0}^{\prime }$, with the vector space of the RBV represented in the *q* − *r* dimension, where *q* is the dimension of the BV and *r* is the rank of the restriction matrix **C**_0_. Therefore, if we want to evaluate the response to restricted selection, such as by a stochastic computer simulation study with known BVs, Equation [Link] can be used as the single criterion for restricted selection. Even if we do not have any true BVs such as practical selection experiment, we can use phenotypes or BVs of BLUP instead of true BVs in Equation [Link]. Equation [Link] can therefore be used as a single criterion for evaluating response to selection with constraints.

## APPENDIX 1

### 1.1

$${\mathrm{AZ}}_{0}^{\prime }{\left(Z_{0}{\mathrm{AZ}}_{0}^{\prime }\right)}^{-1}Z_{0}\mathrm{AJ}=\left[\begin{array}{cc} A_{20} & A_{22}A^{22}A_{21}+A_{22}A^{20}A_{01}+A_{20}A^{02}A_{21}+A_{20}A^{00}A_{01}\\ A_{00} & A_{02}A^{22}A_{21}+A_{02}A^{20}A_{01}+A_{00}A^{02}A_{21}+A_{00}A^{00}A_{01}\\ A_{10} & A_{12}A^{22}A_{21}+A_{12}A^{20}A_{01}+A_{10}A^{02}A_{21}+A_{10}A^{00}A_{01} \end{array}\right]=\left[\begin{array}{cc} A_{20} & A_{21}\\ A_{00} & A_{01}\\ A_{10} & E_{00} \end{array}\right],$$

where $E_{00}=A_{12}A^{22}A_{21}+2A_{12}A^{20}A_{01}+A_{10}A^{00}A_{01}$. Let ${\left[\begin{array}{cc} A_{00} & A_{01}\\ A_{10} & E_{00} \end{array}\right]}^{-1}=\left[\begin{array}{cc} A^{00*} & A^{01}\\ A^{10} & E^{00} \end{array}\right]$.

Since $J^{\prime }{\mathrm{AZ}}_{0}^{\prime }{\left(Z_{0}{\mathrm{AZ}}_{0}^{\prime }\right)}^{-1}Z_{0}\mathrm{AJ}=\left[\begin{array}{cc} A_{00} & A_{01}\\ A_{10} & E_{00} \end{array}\right]$, then,

$${\mathrm{AZ}}_{0}^{\prime }{\left(Z_{0}{\mathrm{AZ}}_{0}^{\prime }\right)}^{-}Z_{0}\mathrm{AJ}{\left[{J^{\prime }\mathrm{AZ}}_{0}^{\prime }{\left(Z_{0}{\mathrm{AZ}}_{0}^{\prime }\right)}^{-}Z_{0}\mathrm{AJ}\right]}^{-}J^{\prime }{\mathrm{AZ}}_{0}^{\prime }{\left(Z_{0}{\mathrm{AZ}}_{0}^{\prime }\right)}^{-}Z_{0}$$

$$=\left[\begin{array}{cc} A_{20} & A_{21}\\ A_{00} & A_{01}\\ A_{10} & E_{00} \end{array}\right]\left[\begin{array}{cc} A^{00*} & A^{01}\\ A^{10} & E^{00} \end{array}\right]\left[\begin{array}{ccc} 0 & I & 0\\ A_{12}A^{22}+A_{10}A^{02} & A_{12}A^{20}+A_{10}A^{00} & 0 \end{array}\right]$$

$$=\left[\begin{array}{ccc} Q_{00} & Q_{01} & 0\\ 0 & I & 0\\ A_{12}A^{22}+A_{10}A^{02} & A_{12}A^{20}+A_{10}A^{00} & 0 \end{array}\right],$$

where

$$Q_{00}=A_{20}A^{01}A_{12}A^{22}+A_{21}E^{00}A_{12}A^{22}+A_{20}A^{01}A_{10}A^{02}+A_{21}E^{00}A_{10}A^{02}$$

and

$$Q_{01}=A_{20}A^{00*}+A_{21}A^{10}+A_{20}A^{01}A_{12}A^{20}+A_{21}E^{00}A_{12}A^{20}+A_{20}A^{01}A_{10}A^{00}+A_{21}E^{00}A_{10}A^{00}.$$

On the other hand,

$${\mathrm{AZ}}_{0}^{\prime }{\left(Z_{0}{\mathrm{AZ}}_{0}^{\prime }\right)}^{-1}Z_{0}=\left[\begin{array}{ccc} I & 0 & 0\\ 0 & I & 0\\ A_{12}A^{22}+A_{10}A^{02} & A_{12}A^{20}+A_{10}A^{00} & 0 \end{array}\right].$$

Therefore,

$$\begin{array}{cc} & {\hat{u}}_{R}=\left[\begin{array}{c} {\hat{u}}_{R2}\\ {\hat{u}}_{R0}\\ {\hat{u}}_{R1} \end{array}\right]=\left[I_{q}⊗\left[\begin{array}{ccc} I & 0 & 0\\ 0 & I & 0\\ A_{12}A^{22}+A_{10}A^{02} & A_{12}A^{20}+A_{10}A^{00} & 0 \end{array}\right]-G_{0}C_{0}{\left(C_{0}^{\prime }G_{0}C_{0}\right)}^{-1}C_{0}^{\prime }⊗\left[\begin{array}{ccc} Q_{00} & Q_{01} & 0\\ 0 & I & 0\\ A_{12}A^{22}+A_{10}A^{02} & A_{12}A^{20}+A_{10}A^{00} & 0 \end{array}\right]\right]\left[\begin{array}{c} u_{2}\\ u_{0}\\ u_{1} \end{array}\right], \end{array}$$

where ${\hat{u}}_{R2}$, ${\hat{u}}_{R0}$, and ${\hat{u}}_{R1}$ are the sub‐vectors of **u**_R_ corresponding to **u**_2_, **u**_0_, and **u**_1_, respectively. When records are no missing, **Q**_00_ = **0** and $Q_{01}=A_{20}A_{00}^{-1}$. Hence

$$u_{R2}=\left[\left[I_{q}-G_{0}C_{0}{\left(C_{0}^{\prime }G_{0}C_{0}\right)}^{-1}C_{0}^{\prime }\right]⊗I\right]E\left(u_{2}|u_{0}\right).$$
